# Overexpression of TRPV6 Inhibits Coronary Atherosclerosis–Related Inflammatory Response and Cell Apoptosis via the PKA/UCP2 Pathway

**DOI:** 10.1155/2024/7053116

**Published:** 2024-10-23

**Authors:** Lei Zheng, Huiying Zhang, Xuewen Li

**Affiliations:** ^1^Department of Cardiovascular Medicine, Third Hospital of Shanxi Medical University, Shanxi Bethune Hospital, Shanxi Academy of Medical Sciences, Tongji Shanxi Hospital, Taiyuan, Shanxi Province 030032, China; ^2^School of Statistics, Shanxi University of Finance and Economics, Taiyuan, Shanxi Province 030006, China

**Keywords:** atherosclerosis, coronary artery disease, inflammatory response, PKA, TRPV6

## Abstract

**Objective:** This research is aimed at unravelling the intricate relationship between transient receptor potential vanilloid 6 (TRPV6), protein kinase A (PKA), uncoupling protein 2 (UCP2), and atherosclerosis. By shedding light on the role of the TRPV6/PKA/UCP2 pathway in inhibiting inflammatory response and cell apoptosis in coronary atherosclerotic plaques, this study provides valuable insights into potential therapeutic targets for treating coronary artery disease (CAD).

**Methods:** We established animal and cell models of atherosclerosis. The expression of TRPV6 was measured using immunohistochemistry and immunofluorescence. Cytokine levels were detected by enzyme-linked immunosorbent assay (ELISA). Cell viability and apoptosis ratio were measured using cell counting kit-8 (CCK-8) and flow cytometry. The binding relationship between TRPV6 and PKA was validated using chromatin immunoprecipitation (CHIP) and coimmunoprecipitation (CoIP). Finally, the expression of the TRPV6/PKA/UCP2 signaling pathway and apoptosis-related factors was detected using western blot (WB) and quantitative real-time polymerase chain reaction (qRT-PCR).

**Results:** TRPV6 was significantly decreased in atherosclerosis mouse and cell model. CHIP and CoIP assays indicated that TRPV6 binds to PKA and positively regulated its expression in oxidized low-density lipoprotein (ox-LDL)–treated human umbilical vein endothelial cells (HUVECs). Overexpression of TRPV6 significantly increased cell viability and inhibited apoptosis, whereas silencing TRPV6 had the opposite effect. Additionally, the overexpression of TRPV6 remarkably declined the expression of tumor necrosis factor-alpha (TNF-*α*), interleukin-6 (IL-6), and interleukin-1 beta (IL-1*β*). However, after silencing PKA, this effect was partially reversed, the cell viability and inflammatory response remarkably enhanced, and apoptosis significantly declined in oe-TRPV6 + si-PKA group.

**Conclusions:** In summary, our study demonstrated that TRPV6 inhibited apoptosis and inflammatory response in the atherosclerosis cell model through the regulation of the PKA/UCP2 pathway.

## 1. Introduction

Coronary artery disease (CAD) is the leading cause of global mortality and disability-adjusted life years (DALYs) [1]. The formation of coronary atherosclerotic plaques is the primary cause of CAD [2–4]. Now, emerging evidence has highlighted the potential role of TRP family in modulating the inflammatory response and cell apoptosis in atherosclerotic plaques [5, 6].

Transient receptor potential vanilloid 6 (TRPV6) has gained attention due to its involvement in calcium transport and cellular homeostasis. TRPV6 activation is involved in cell proliferation as well as apoptosis [7, 8]. In our previous clinical study, we found that lower levels of TRPV6 were associated with shorter survival times [9]. TRPV6 is involved in various pathological processes that engage multiple signaling pathways, including CaM, CaMKII, CaMKK, PI3K/Akt/Gsk-3*β*, and MAPK/JNK [10–12], many of which are implicated in the pathophysiology of coronary atherosclerosis. However, to date, no research has investigated the role and mechanisms of TRPV6 in coronary atherosclerosis. Protein kinase A (PKA) is a vital signaling molecule involved in multiple intracellular pathways, regulating cell survival, inflammation, and apoptosis [13, 14]. Activation of PKA is protective against atherosclerosis [15]. However, the specific mechanisms underlying its action in atherosclerotic plaques remain incompletely understood. Uncoupling protein 2 (UCP2) is a mitochondrial protein known for its role in energy metabolism and reducing reactive oxygen species (ROS) production. UCP2 has demonstrated anti-inflammatory and antiapoptotic effects in various diseases, possibly involved in the development of atherosclerosis [16, 17].

Based on the existing knowledge of TRPV6, PKA, and UCP2, this study is aimed at investigating the potential interplay between these molecules in the context of coronary atherosclerotic plaques. We hypothesize that activation of the TRPV6/PKA/UCP2 pathway may exert inhibitory effects on the inflammatory response and cell apoptosis within these plaques. To address this hypothesis, we measured the expression and activity of TRPV6, PKA, and UCP2. Additionally, we assessed the levels of inflammatory mediators and apoptotic markers in response to modulation of this pathway. This research is aimed at unravelling the intricate relationship between TRPV6, PKA, UCP2, and atherosclerosis. By shedding light on the role of the TRPV6/PKA/UCP2 pathway in inhibiting inflammatory response and cell apoptosis in coronary atherosclerotic plaques, this study provides valuable insights into potential therapeutic targets for treating CAD.

## 2. Methods

### 2.1. Animal Model

C57BL/6 male apolipoprotein E-deficient (apoE^−/−^) mice (5 weeks old, Beijing Vital River Laboratory Animal Technology) were housed in a suitable environment (20–26°C, 60% humidity, 12-h light/dark cycle) with ad libitum access to food and water. After a 1-week acclimatization period, the experimental group of mice was started on a standard diet of 21% fat, 2% cholesterol, and 5% lard to induce atherosclerosis. Eighteen apoE^−/−^ mouse controls were fed a normal diet [18]. At the end of the 12th week, 12 apoE^−/−^ mice (six from the HFD group and six from the normal diet group) were euthanized after collecting serum samples, and the aortic sinuses were excised. This study adhered to the international guidelines for animal research projects and obtained approval from the institutional animal ethics committee of Shanxi Bethune Hospital (No. 2023075).

### 2.2. Oil Red O Stain

To assess the formation of atherosclerotic plaque lesions, aortic sinus sections were dehydrated in 100% isopropanol for 5 min and then stained with 0.5% oil red O (Sigma-Aldrich, United States). Computer-assisted image quantification was performed using Image-Pro Plus 6.0.

### 2.3. Immunohistochemistry (IHC)

Aortic sinus tissue sections from euthanized mice were fixed in 4% paraformaldehyde, dehydrated, embedded in paraffin, and sectioned into consecutive 4 *μ*m slices. Deparaffinization was performed using routine methods. Subsequently, tissue sections were incubated overnight at 4°C with the corresponding primary antibodies. The tissues were then incubated with the appropriate secondary antibodies for 30 min at 37°C. Following this, the tissues were incubated with HRP-conjugated working solution and stained with 3,3⁣′-diaminobenzidine for 5–10 min. The staining time was adjusted under a microscope. After counterstaining with hematoxylin for 1 min, the tissues were fixed with mounting medium, dried, and photographed. Five high-power fields were selected for observation and counting under a bright-field microscope. Protein with cytoplasmic green staining was considered positive, indicating the expression of TRPV6 in each group of mice.

### 2.4. Enzyme-Linked Immunosorbent Assay (ELISA)

Serum samples were centrifuged at 2000 × *g* for 15 min using an Eppendorf 5804R refrigerated centrifuge (Eppendorf AG, Germany) and then stored at −80°C for freezing until further analysis. ELISA kits (MyBioSource, United States) were utilized to measure expression of TRPV6, TNF-*α*, IL-6, and IL-1*β*, strictly following the provided instructions.

### 2.5. Cell Culture and Treatment

Human umbilical vein endothelial cells (HUVECs; ATCC, Manassas, Virginia) were cultured in RPMI-1640 medium at 37°C and 5% CO^2^ that was supplemented with 10% Gibco fetal bovine serum (FBS) and 100 *μ*g/mL penicillin-streptomycin. Atherosclerotic cell models were established by seeding 3 × 10^5^ logarithmically grown HUVEC in 6-well plates and treated with 50 *μ*g/mL of oxidized low-density lipoprotein (ox-LDL) for 24 h [19].

### 2.6. Cell Transfection

The siRNAs (si-TRPV6#1, si-TRPV6#2, si-PKA#1, and si-PKA#2), TRPV6 overexpression plasmids (oe-TRPV6#1 and oe-TRPV6#2), and the negative control were procured from GeneChem Corp. for use in cell transfection experiments.

For transfection, cells were transfected with the above siRNAs or plasmids using Lipofectamine 3000 (Invitrogen, CA, United States). Briefly, synchronized growing cells were detached using a sterile cell scraper and resuspended in cell suspension. The cells were then seeded into the 6-well plates. After 24 h of incubation, 4 *μ*L/well of Lipofectamine and 4 *μ*L/well of each vector were mixed and incubated at room temperature for 10 min, followed by approximately 30 min of incubation. The mixture was then added to the cells and thoroughly mixed and incubated for 48 h.

### 2.7. Immunofluorescence (IFC)

Cells were fixed, permeabilized, and incubated overnight at 4°C with anti-TLR9 antibody (1:200, Sigma-Aldrich) and anti-MyD88 antibody (1:100, Sigma-Aldrich). Subsequently, cells were incubated with corresponding secondary antibodies at room temperature for 1 h. DAPI was used for nuclear staining.

### 2.8. Cell Counting Kit-8 (CCK-8)

Plate the cells in 96-well plates at a density of 5 × 10^3^cells/well. After incubating the cells with CCK-8 reagent (10 *μ*L, Sangon Biotech) for 2 h, measure the absorbance at 450 nm using a microplate spectrophotometer to determine cell proliferation at different time points. Calculate the IC_50_ concentration.

### 2.9. Flow Cytometry for Cell Apoptosis

After the incubation of 24 h, HK-2 cells were collected into 1.5 mL tubes with annexin V-FITC and propidium iodide (PI) regents, following with incubation of 10 min. To detect cell apoptosis, 200 *μ*L PI supplemented with 1 mL phosphate-buffered saline (PBS) was added into the flow tube. Then, the apoptosis was measured using flow cytometry.

### 2.10. Chromatin Immunoprecipitation (CHIP)

HUVEC supernatants were treated with respective antibodies. Then, 60 *μ*L of protein A-sepharose/salmon sperm DNA mixture was added and incubated at 4°C for 2 h. After centrifugation at 700 rpm for 1 min, the supernatant was removed, and the beads were washed twice with 1 mL each of low-salt buffer, high-salt buffer, lithium chloride solution, and TE buffer with trace elements. The beads were then eluted twice with 250 *μ*L of CHIP wash buffer, and crosslinking was reversed by adding 20 *μ*L of 5 M sodium chloride. DNA was extracted, and the promoter sequences in the complex were quantified using fluorescence quantitative PCR to validate the regulatory relationship between TRPV6 and PKA.

### 2.11. Coimmunoprecipitation (CoIP)

Cell pellets, washed with PBS, were incubated with corresponding vectors in PBS. Crosslinking was performed at room temperature for 30 min, followed by 2 h of rotation at 4°C. After washing with PBS, crosslinking was quenched in a three-buffer saline (pH 7.0) solution and incubated at 4°C for 20 min. Cell lysis was performed in CoIP buffer containing 20 mM Tris-HCl (pH 8.0), 100 mM NaCl, 1 mM EDTA, and 0.5% IGEPAL CA-630 and supplemented with a protease inhibitor cocktail. Subsequently, the lysate was incubated for 20 min at 4°C on a rotating platform. A mixture of 1.2 mg of lysate and 2.5 *μ*g of the corresponding antibody was incubated overnight at 4°C. In the lysate-antibody mixture, 20 *μ*L of BSA-blocked Protein G Dynabeads (Invitrogen, 100-03D) were added and incubated at 4°C for 3 h. The beads were washed three times with PBS, boiled in Laemmli loading buffer at 95°C for 5 min, and the protein-antibody complexes were eluted. Fifteen micrograms of cell lysate or 5 *μ*L of immunoprecipitated material was separated by SDS-PAGE. WB analysis was performed using the corresponding primary antibody to detect the interaction between TRPV6 and PKA.

### 2.12. Quantitative Real-Time Polymerase Chain Reaction (qRT-PCR)

To obtain RNA from NP cells and tissues, we utilized the RNAiso Plus kit (procured from Takara, Japan) and subsequently performed reverse transcription into cDNA using the PrimeScript one-step qRT-PCR kit (obtained from Takara). For RT-qPCR, we employed the SYBR Premix Ex Taq (TaKaRa) and the ABI PRISM7300 Sequence Detection System (from Applied Biosystems). Primer sequences were as follows: TRPV6, forward 5⁣′-GAGCCGAGACGAGCAGAAC-3⁣′and reverse 5⁣′-TGGACATCGTTTTCTTTGGCAG-3⁣′; PKA, forward 5⁣′-CCAGACTCGGATCGCAAATGA-3⁣′and reverse 5⁣′-AGAATGTTGTCCGGTCTTTTCAG-3⁣′; UCP2, forward 5⁣′-ATGGTTGGTTTCAAGGCCACA-3⁣′and reverse 5⁣′-TTGGCGGTATCCAGAGGGAA-3⁣′; and GAPDH, forward 5⁣′-CACCCACTCCTCCACCTTTG-3⁣′and reverse 5⁣′-CCACCACCCTGTTGCTGTAG-3⁣′. GAPDH was served as an internal control. The mRNA expressions were calculated by the 2^−ΔΔCt^ method.

### 2.13. Western Blot (WB)

Total proteins were extracted from both the cells and lysates of aortic sinus tissue sections. The Pierce BCA Protein Assay Kit (Thermo Scientific) was used for protein quantification. Subsequently, 20 *μ*g of the extracted proteins was separated using SDS-PAGE and transferred to PVDF membranes. Following overnight incubation with a primary antibody at 4°C and subsequent washing, the membranes were incubated with goat anti-rabbit IgG H&L secondary antibody (ab96899, 1/1000, Abcam) at 37°C for 45 min. The primary antibodies included TRPV6 antibody (ab238107, 1/500, Abcam), PKA antibody (ab75991, 1/1000, Abcam), UCP2 antibody (ab67241, 1/1000, Abcam), bcl-2 antibody (ab32124, 1/1000, Abcam), bax antibody (ab32503, 1/1000, Abcam), cleaved caspase-3 antibody (ab32042, 1/5000, Abcam), GAPDH antibody (ab8245, 1/1000, Mybiosource), and GAPDH antibody (Abcam, United States). To visualize the protein bands, we utilized the Pierce ECL Western Blotting Substrate, which was procured from Pierce in Shanghai, China. The membranes were developed and imaged using a chemiluminescence imaging system.

### 2.14. Statistical Analysis

The data is presented as mean ± SD, and we utilized one-way ANOVA followed by a Tukey post hoc test for statistical comparisons. For significance, a *p* value less than 0.05 was considered.

## 3. Results

### 3.1. Decreased Expression of TRPV6 in Atherosclerosis Mice

**Figure 1:**
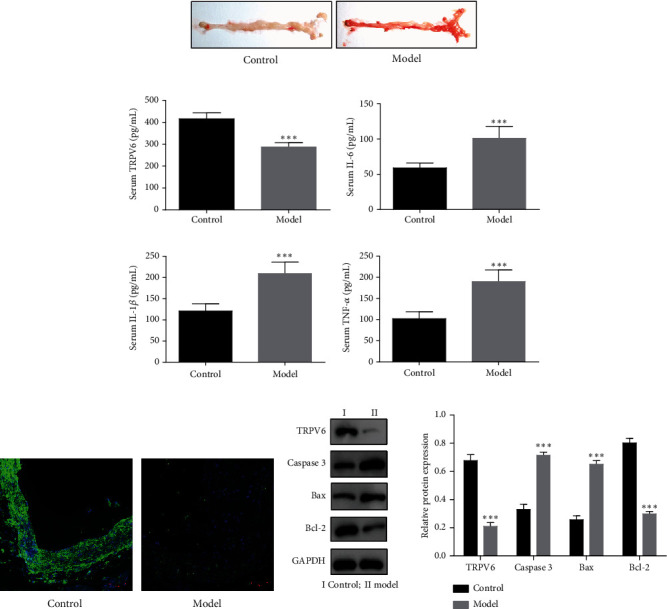
The expression of transient receptor potential vanilloid 6 (TRPV6) was significantly decreased in atherosclerosis mouse model. (a) Oil red O staining on mouse aortic sinus tissue sections. (b) Serum TRPV6, interleukin-6 (IL-6), interleukin-1 beta (IL-1*β*), and tumor necrosis factor-alpha (TNF-*α*) levels in atherosclerosis mouse model. (c) Immunohistochemical (IHC) detection of TRPV6 expression. (d) The protein expression of TRPV6, cleaved caspase-3, B cell lymphoma 2 (Bcl-2)–associated X protein (Bax), and B cell lymphoma 2 (Bcl-2) was measured by western blot (WB). Continuous data between two groups were compared using Student's *t*-test, while comparisons among three or more groups were analyzed by one-way analysis of variance and Tukey's post hoc test. ⁣^∗∗∗^*p* < 0.001 compared with the control group.

For the expression of TRPV6 in atherosclerosis mice, we performed oil red O staining on mouse aortic sinus tissue sections. As shown in Figure 1(a), compared to the almost nonstained area, the atherosclerosis mice exhibited dense and enriched red staining, indicating extensive lesion areas and successful model establishment. In addition, serum TRPV6 levels were remarkably declined, while serum TNF-*α*, IL-6, and IL-1*β* levels were remarkably elevated (Figure 1(b)). Furthermore, IHC and WB results also demonstrated a significant decrease of TRPV6 in the mouse aortic sinus tissue sections of the model group (Figures 1(c) and 1(d)). In addition, we also found that the atherosclerosis mice exhibited significant increases in bax and cleaved caspase-3.

### 3.2. Expression of TRPV6, PKA, and UCP2 in Atherosclerosis Cell Model

**Figure 2:**
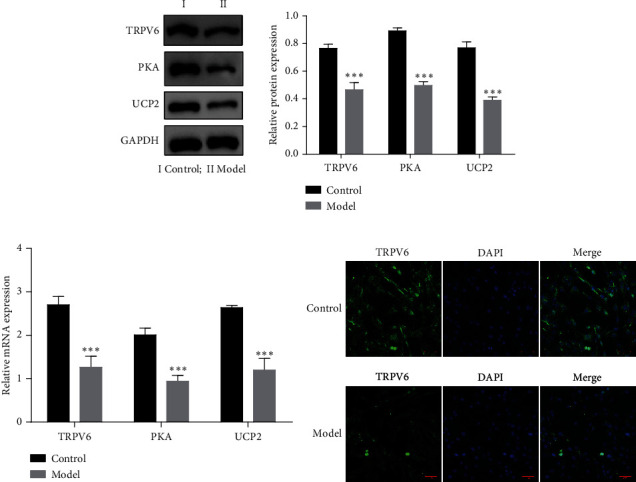
The expression of transient receptor potential vanilloid 6 (TRPV6), protein kinase A (PKA), and uncoupling protein 2 (UCP2) in atherosclerosis cell model. The protein expression of TRPV6, PKA, and UCP2 was measured by western blot (WB) (a) and quantitative polymerase chain reaction (qPCR) (b). (c) Immunofluorescence (IFC) detection of TRPV6 expression. Continuous data between two groups were compared using Student's *t*-test, while comparisons among three or more groups were analyzed by one-way analysis of variance and Tukey's post hoc test. ⁣^∗∗∗^*p* < 0.001 compared with the control group.

Subsequently, we treated HUVECs with 50 *μ*g/mL of ox-LDL for 24 h to establish an atherosclerosis cell model. Consistent with the trends observed in the animal model, WB and PCR results showed that TRPV6, PKA, and UCP2 expression in the ox-LDL-treated cells decreased remarkably (Figures 2(a) and 2(b)). IFC experiments demonstrated that the expression of TRPV6 was decreased significantly in the atherosclerosis cell model (Figure 2(c)).

### 3.3. Overexpression of TRPV6 Inhibits Apoptosis and Inflammatory Response

**Figure 3:**
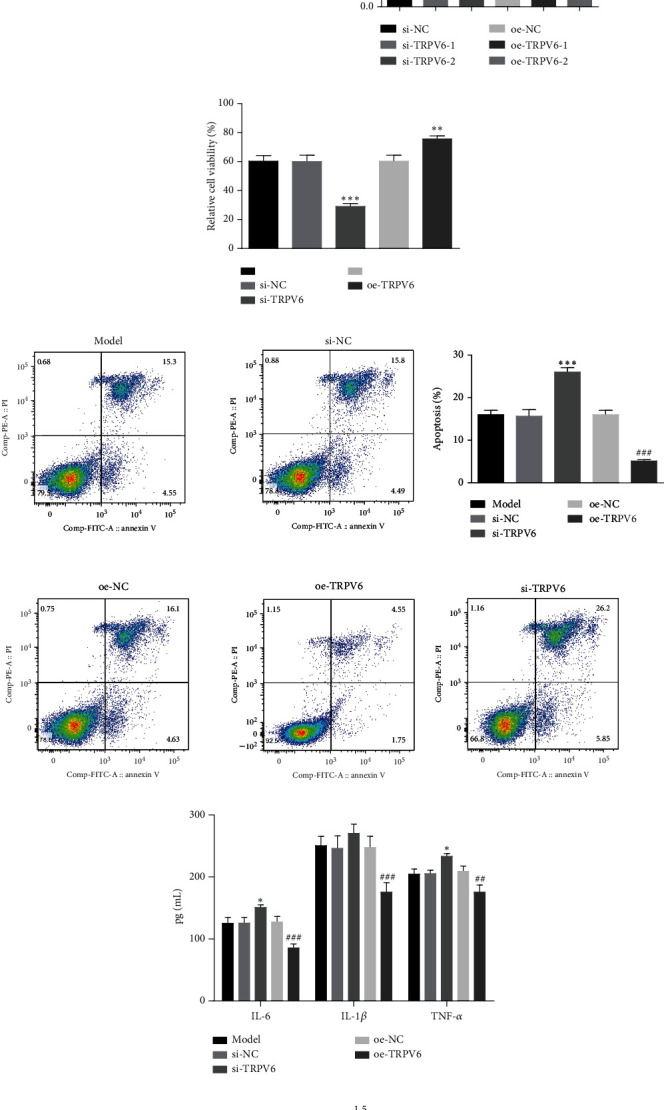
Overexpression of transient receptor potential vanilloid 6 (TRPV6) inhibits apoptosis and inflammatory response. (a) The protein expression of TRPV6 was measured by western blot (WB) and quantitative polymerase chain reaction (qPCR). (b) Cell counting kit-8 (CCK-8) was used to measure cell viability. (c) Flow cytometry was used to measure apoptosis. (d) Enzyme-linked immunosorbent assay (ELISA) was used to measure the expression of interleukin-6 (IL-6), interleukin-1 beta (IL-1*β*), and tumor necrosis factor-alpha (TNF-*α*). (e) WB was used to measure the expression of cleaved caspase-3, B cell lymphoma 2 (Bcl-2)–associated X protein (Bax), and B cell lymphoma 2 (Bcl-2). Continuous data between two groups were compared using Student's *t*-test, while comparisons among three or more groups were analyzed by one-way analysis of variance and Tukey's post hoc test. ⁣^∗^*p* < 0.05, ⁣^∗∗^*p* < 0.01, and ⁣^∗∗∗^*p* < 0.001 compared with the silencing negative control (si-NC) group. ^#^*p* < 0.05, ^##^*p* < 0.01, and ^###^*p* < 0.001 compared with the overexpression negative control (oe-NC) group.

We further investigated the role of TRPV6 in atherosclerosis by transfecting ox-LDL-treated cells with siRNAs (si-TRPV6#1 and si-TRPV6#2) and TRPV6 overexpression plasmids (oe-TRPV6#1 and oe-TRPV6#2). The results of PCR indicated that si-TRPV6 remarkably knocked down the expression of TRPV6, while oe-TRPV6 significantly increased TRPV6 expression (Figure 3(a)). We selected si-TRPV6#1 and oe-TRPV6#2, which had better transfection efficiency, for subsequent experiments. The overexpression of TRPV6 significantly increased cell viability and inhibited apoptosis, whereas silencing TRPV6 had the opposite effect (Figures 3(b) and 3(c)). ELISA experiments also demonstrated that the overexpression of TRPV6 significantly suppressed the inflammatory response in ox-LDL-treated HUVECs (Figure 3(d)). Additionally, the oe-TRPV6 group showed a significant increase of bcl-2 levels, while bax and cleaved caspase-3 levels were significantly decreased (Figure 3(e)). These findings collectively indicated that the overexpression of TRPV6 could inhibit cell apoptosis and the inflammatory response in ox-LDL-treated HUVECs.

### 3.4. TRPV6 Bound to PKA and Positively Regulated PKA

**Figure 4:**
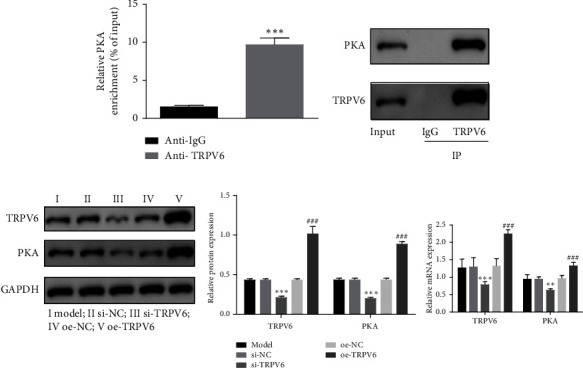
Transient receptor potential vanilloid 6 (TRPV6) bound to protein kinase A (PKA) and positively regulated PKA. Chromatin immunoprecipitation (CHIP) (a) and coimmunoprecipitation (COIP) (b) were performed to verify the binding relationship between TRPV6 and PKA. (c) The expression of TRPV6 and PKA was measured by western blot (WB) and quantitative polymerase chain reaction (qPCR). Continuous data between two groups were compared using Student's *t*-test, while comparisons among three or more groups were analyzed by one-way analysis of variance and Tukey's post hoc test. ⁣^∗^*p* < 0.05, ⁣^∗∗^*p* < 0.01, and ⁣^∗∗∗^*p* < 0.001 compared with the silencing negative control (si-NC) group. ^#^*p* < 0.05, ^##^*p* < 0.01, and ^###^*p* < 0.001 compared with the overexpression negative control (oe-NC) group.

To further investigate the mechanism of action of TRPV6 and PKA in the atherosclerosis cell model, we employed CHIP and CoIP assays to explore the interaction and regulatory relationship between TRPV6 and PKA. As shown in Figure 4(a), CHIP results demonstrated a significant increase in the enrichment level of PKA in the anti-TRPV6 group compared to the anti-IgG group in ox-LDL-treated HUVECs, indicating a specific binding between TRPV6 and PKA. CoIP experiments also confirmed the interaction between TRPV6 and PKA (Figure 4(b)). Silencing TRPV6 significantly decreased the expression of PKA, while the overexpression of TRPV6 significantly enhanced the expression of PKA (Figure 4(c)). This suggested that TRPV6 binds to PKA and positively regulated its expression in ox-LDL-treated HUVECs.

### 3.5. TRPV6 Inhibited Apoptosis and Inflammatory Response via Regulation of PKA/UCP2

**Figure 5:**
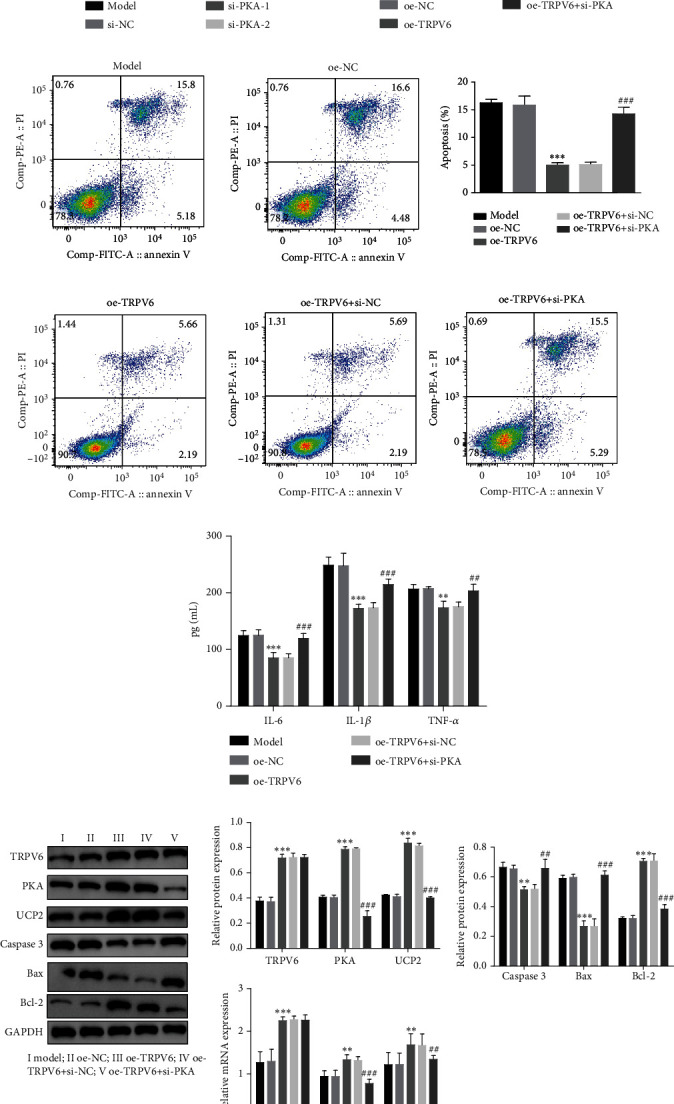
Transient receptor potential vanilloid 6 (TRPV6) inhibited apoptosis and inflammatory response via regulation of protein kinase A (PKA)/uncoupling protein 2 (UCP2). (a) The expression of PKA was measured by quantitative polymerase chain reaction (qPCR). (b) Cell counting kit-8 (CCK-8) was used to measure cell viability. (c) Flow cytometry was used to measure apoptosis. (d) Enzyme-linked immunosorbent assay (ELISA) was used to measure the expression of interleukin-6 (IL-6), interleukin-1 beta (IL-1*β*), and tumor necrosis factor-alpha (TNF-*α*). (e) Western blot (WB) and qPCR were used to measure the expression of TRPV6, PKA, UCP2, cleaved caspase-3, B cell lymphoma 2 (Bcl-2)–associated X protein (Bax), and B cell lymphoma 2 (Bcl-2). Continuous data between two groups were compared using Student's *t*-test, while comparisons among three or more groups were analyzed by one-way analysis of variance and Tukey's post hoc test. ⁣^∗^*p* < 0.05, ⁣^∗∗^*p* < 0.01, and ⁣^∗∗∗^*p* < 0.001 compared with the overexpression negative control (oe-NC) group. ^#^*p* < 0.05, ^##^*p* < 0.01, and ^###^*p* < 0.001 compared with the overexpression TRPV6 + silencing negative control (oe-TRPV6 + si-NC) group.

We further investigated the impact of inhibiting PKA expression on cell apoptosis and inflammatory response in the atherosclerosis cell model. si-PKA effectively knocked down the expression level of PKA (Figure 5(a)). The overexpression of TRPV6 significantly increased cell viability and reduced the proportion of apoptotic cells. However, after silencing PKA, this effect was partially reversed. The oe-TRPV6 + si-PKA group showed a significant increase in apoptosis and a significant decrease in cell viability (Figures 5(b) and 5(c)). Additionally, ELISA results indicated that the overexpression of TRPV6 remarkably suppressed the inflammatory response in ox-LDL-treated HUVECs, while silencing PKA enhanced the inflammatory response. Specifically, the oe-TRPV6 + si-PKA group showed remarkably enhanced levels of cytokines (Figure 5(d)). Furthermore, as shown in Figure 5(e), the overexpression of TRPV6 remarkably enhanced the expression of PKA, UCP2, and the apoptosis-associated factor bcl-2, while decreasing the expression of caspase-3 and bax. As we expected, this was reversed after silencing PKA, and the expression of PKA, UCP2, and bcl-2 was reduced, while the expression of caspase-3 and bax was rebounded. These results suggested that TRPV6 inhibited apoptosis and inflammatory responses by regulating PKA/UCP2 in an atherosclerotic cell model.

## 4. Discussion

Worldwide, CAD causes approximately 17.8 million deaths annually. Moreover, in developed countries, the treatment of CAD imposes a significant economic burden, with reported healthcare costs exceeding $200 billion annually in the United States [20]. Here, we demonstrated that the expression of TRPV6 was significantly decreased in both in vivo and in vitro models of atherosclerosis. We further demonstrated that TRPV6 inhibited inflammatory response and apoptosis in atherosclerosis through the regulation of PKA/UCP2.

The initial stage of atherosclerosis involves endothelial dysfunction, which leads to the recruitment and adhesion of monocytes to the arterial wall. As a consequence, these monocytes undergo differentiation into macrophages and engulf ox-LDL, leading to the formation of foam cells [21, 22]. The persistent presence of foam cells triggers an inflammatory response with the release of proinflammatory cytokines [23]. The inflammatory environment promotes smooth muscle cell migration, proliferation, and the formation of a fibrous cap over the lipid-rich plaque, but plaque instability can lead to rupture, releasing proapoptotic factors and causing cell apoptosis [24]. Conversely, the process of cell apoptosis within the plaque can further exacerbate inflammation, as it contributes to the release of matrix metalloproteinases and the formation of a necrotic core [25]. In our study, both in vivo and in vitro models exhibited a significant increase in the levels of cytokines, as well as apoptotic factors. However, following the overexpression of TRPV6, the inflammatory response and apoptosis was suppressed.

The upregulation of TRPV6, on the other hand, is associated with cancer progression, as it contributes to promoting cell proliferation and inhibiting cell apoptosis, and it is overexpressed in a variety of human cancers [26, 27]. Additionally, studies have reported that TRPV6 can regulate calcium ion balance and pancreatic inflammation in pancreatitis [28]. In our clinical research, we have also found that patients with lower serum TRPV6 levels, such as those with acute myocardial infarction, have higher levels of CK-MB and NT-pro-BNP [9]. In other members of the TRP family, Marshall et al. found that TRPV1KO mice exhibited protective effects against obesity-induced hypertension, mild inflammation, and impaired glucose tolerance [29]. Randhawa and Jaggi demonstrated that the TRPV4 channel played a crucial regulatory role in various biological processes, including the development of pulmonary edema in heart failure and blood flow–induced atherosclerosis [30]. The latest reviews also indicate that an increasing number of studies are targeting TRPV1 and TRPV2 channels as therapeutic targets for cardiovascular diseases [31]. However, limited research has focused on the impact of TRPV6 on inflammatory response and cell apoptosis in cardiovascular diseases. Our study contributed to filling this gap and validates in vitro experiments showing that the overexpression of TRPV6 significantly increases cell viability, reduced the proportion of apoptotic cells, and suppressed inflammatory response. Conversely, silencing TRPV6 produced the opposite effects. Further mechanistic studies have revealed that TRPV6 regulates the PKA/UCP2 signaling pathway, thus modulating inflammatory response and cell apoptosis in atherosclerosis. The PKA/UCP2 signaling pathway regulates inflammatory response and apoptosis in various diseases [32, 33]. Recent research also demonstrates that activation of TRPV1 can improve mitochondrial dysfunction and enhance the UCP2/PKA pathway to counteract ROS-mediated coronary artery lesions [34]. Our findings suggest that TRPV6, through the regulation of the same downstream signaling pathway, PKA/UCP2, inhibited apoptosis and inflammatory response in the atherosclerosis cell model.

The limitation of this study is that firstly, it remains unclear whether TRPV6 is regulated by other genes. Secondly, we did not investigate the impact of overexpressing TRPV6 on the progression of atherosclerotic plaques in animal studies. Thirdly, in this study, our primary focus was to investigate the role of TRPV6 in inflammation and cellular apoptosis in an atherosclerotic cell model, without exploring the effects of other TRPV family proteins. Despite these limitations, the findings from this study have significant clinical implications. Targeting the TRPV6/PKA/UCP2 pathway may offer a novel therapeutic strategy for managing coronary atherosclerotic plaques.

## 5. Conclusions

Our study highlights the pivotal role of TRPV6 in mitigating apoptosis and inflammatory responses in atherosclerosis cell models via modulation of the PKA/UCP2 signaling pathway. By demonstrating that TRPV6 overexpression can enhance cell viability and suppress proinflammatory cytokines, we provide a foundation for the potential development of TRPV6-targeted therapies.

## Data Availability

The data that support the findings of this study are available from the corresponding author upon a reasonable request.
